# Mutations in the dimer interfaces of the dengue virus capsid protein affect structural stability and impair RNA-capsid interaction

**DOI:** 10.1038/s41598-019-39185-3

**Published:** 2019-02-26

**Authors:** Janaina Figueira-Mansur, Estefania A. Aguilera, Rafael M. Stoque, Gustavo T. Ventura, Ronaldo Mohana-Borges

**Affiliations:** Laboratório de Genômica Estrutural, Instituto de Biofísica Carlos Chagas Filho, Universidade Federal do Rio de Janeiro, Rio de Janeiro, Brazil

## Abstract

The dengue virus 2 capsid protein (DENV2C) plays a primary structural role in the protection of the viral genome and is crucial for nucleocapsid assembly. In this study, we generated single mutants of DENV2C at L50 and L54 residues of the α2 helix, which was shown to interfere with the integration of the capsid into lipid droplets, and at residues L81 and I88 located in the α4 helix, which was shown to affect viral assembly. We demonstrated that the oligomeric states of DENV2C and its mutants exist primarily in the dimeric state in solution. All single-point mutations introduced in DENV2C promoted reduction in protein stability, an effect that was more pronounced for the L81N and I88N mutants, but not protein unfolding. All the single-point mutations affected the ability of DEN2C to interact with RNA. We concluded that mutations in the α2-α2′ and α4-α4′ dimer interfaces of DENV2C affect the structural stability of the protein and impair RNA-capsid interaction. These effects were more pronounced for mutations at the L81 and I88 residues in the α4 helix. These results indicate the importance of the α4-α4′ dimer interface, which could be studied as a potential target for drug design in the future.

## Introduction

Dengue virus (DENV) is member of the *Flavivirus* genus, which belongs to the *Flaviviridae* family and includes Zika virus, yellow fever virus, Japanese encephalitis virus and West Nile virus, all of which cause mosquito-borne human infections^1^. The occurrence of dengue worldwide has grown in recent decades, and the latest estimates note an incidence of 390 million dengue infections annually, with 96 million presenting clinical manifestations^2,3^. This disease has spread to new areas and is currently present in approximately 128 countries, with an estimate of 3.9 billion people living in areas with risk of dengue infection^2,4^.

DENV is a positive-sense single-stranded RNA (ssRNA) virus that has an icosahedral structure and is approximately 50 nm in diameter. The genome of DENV, which is approximately 10.7 kb in size, is translated to a polypeptide that is cleaved by viral and host proteases to generate three structural (envelope [E], pre-membrane [prM] and capsid [C]) and seven non-structural (NS1, NS2A, NS2B, NS3, NS4A, NS4B and NS5) proteins^5,6^. Non-structural proteins are involved in genome replication, viral assembly and modulation of host immune responses^6,7^. The capsid protein associates with the genomic RNA to form the viral nucleocapsid, which is enveloped by a lipid bilayer containing embedded E and M proteins, constituting the viral particle^8^.

The capsid protein is a highly basic dimeric protein composed of 100 amino acid residues. A key function of the capsid protein is genome encapsidation during viral assembly. The homodimeric structure of DENV2C was solved by nuclear magnetic resonance (NMR); each monomer is composed of four α-helices (α1 to α4) that are connected by loops, with the roughly twenty N-terminal amino acid residues predicted as being largely unstructured in solution^9–13^. The structure of the symmetric homodimer of DENV2C has a large dimerization surface, with two pairs of antiparallel helical interfaces, namely, α2–α2′ and α4–α4′, that are stabilized by hydrophobic interactions. Residues of the α1 and α3 helices of the dimer form a concave hydrophobic cleft. Based on the differential charge distribution of the capsid and the shape of hydrophobic cleft, it was proposed that the α4–α4′ region, which is rich in basic residues, interacts with RNA, and the α2–α2′ region located at the bottom of the hydrophobic cleft interacts with membranes^10^.

The capsid protein was shown to accumulate in the cytoplasm of DENV-infected cells at the boundaries of lipid droplets, which are organelles derived from the endoplasmic reticulum^12,14,15^. An increase in the number of lipid droplets per cell was also observed during DENV infection, suggesting a relation between viral replication and lipid-droplet metabolism. This relation was reinforced by the fact that pharmacological intervention of lipid droplet production led to decreased viral replication^15^. An internal hydrophobic domain of DENV4C (aa 45 to 65) was previously demonstrated to be important for the mediation of capsid integration into membranes^16^. Mutagenesis assays using infectious DENV clones also identified specific hydrophobic residues in the previously described hydrophobic region of the capsid protein that are essential for the association of the capsid protein to lipid droplets. In addition, mutations of residues L50 and L54 of DENVC can interfere with capsid integration into lipid droplets and can impair viral particle assembly. It has been suggested that lipid droplets are able to sequester DENVC early during infection and act as a scaffold for genome encapsidation^15^. Conserved residues in the unstructured N-terminal region, such as a 10-amino acids crucial motif behind a peptide inhibitor of DENVC interaction with host lipid droplets (pep14–23), as well as specific residues in the α2–α2′ dimer interface, namely amino acids 51 to 54, were demonstrated, via mapping, to be affected by the interaction with lipid droplets^12,13,16^.

Despite recent advances, the biological importance of the capsid protein in DENV infection, and thus in DENV pathogenesis, has not yet been fully elucidated. Little is known about structural stability of the capsid protein or about the interaction of this protein with RNA molecules. To tackle these questions, we reproduced mutations at residues 50 and 54 in the α2 helix of DENV2C that have already been shown to interfere with the integration of the capsid into lipid droplets^15^; we also reproduced mutations at residues 81 and 88 in the α4 helix, which have been described by Patkar *et al*. as affecting viral assembly^17^. We evaluated the structural stability and the oligomeric states of DENV2C and its single-point mutants (L50S, L54S, L81N and I88N). We also evaluated the effects of these mutations on RNA-capsid interaction. Our findings highlight the importance of the α2–α2′ and α4–α4′ dimer interfaces of the DENV2C protein; mutations in these helices can affect the structural stability of the protein and decrease RNA-capsid affinity.

## Results

### Mutations in the α2–α2′ and α4–α4′ dimer interfaces affect the secondary structure of the DENV2C protein

**Figure 1:**
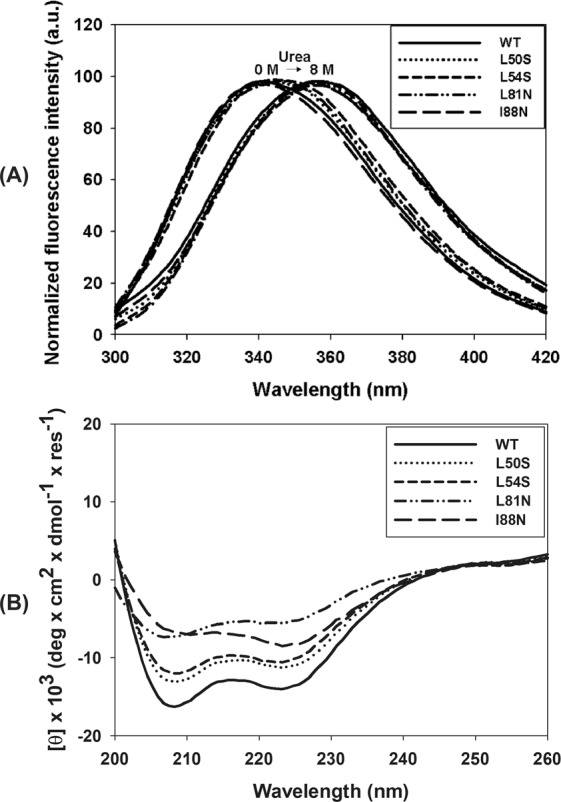
Analysis of the secondary and tertiary structures of WT and single-point mutant (L50S, L54S, L81N and I88N) DENV2C proteins by tryptophan fluorescence spectroscopy and circular dichroism. (**A**) Tryptophan fluorescence spectra were obtained by diluting these proteins to a final concentration of 5 µM in 50 mM sodium phosphate buffer (pH 6.0)/0.2 M NaCl. Protein unfolding was evaluated by the addition of urea to final concentration of 8 M. (**B**) CD spectra were obtained in a Chirascan (Applied Photophysics, United Kingdom) using quartz cuvettes with a 0.01-cm path length at 25 °C. The proteins were diluted in 50 mM sodium phosphate buffer (pH 6.0)/200 mM NaCl to a final concentration of 30 µM. Final spectra were the averages of triplicates after buffer and baseline subtractions and were plotted from wavelengths of 200 nm to 260 nm.

The WT DENV2C and its single-point mutants L50S, L54S, L81N and I88N were expressed and purified at high yields. Analysis of their secondary and tertiary structures by CD and tryptophan fluorescence spectroscopies, respectively, demonstrated that the proteins were properly folded (Fig. 1). The maximum-emission peaks in the tryptophan fluorescence spectra ranged from 340 to 346 nm for all proteins. The maximum-emission peaks of the spectra of all proteins shifted to approximately 356 nm in the presence of 8 M urea, suggesting that this condition promoted complete denaturation of the WT and mutant DENV2C proteins (Fig. 1A). The CD spectra of the proteins showed negative peaks at approximately 208 and 222 nm, which is typical for α-helical proteins (Fig. 1B) and was expected for DENV2C, whose 3D structure solved by NMR demonstrated that this protein is composed of 4 α-helices^10^. However, the mutations significantly affected the secondary structure of the DENV2C protein. The mutations L50S and L54S led to a decrease of approximately 18% in the CD signals from both proteins compared to the signal from the WT protein. Curiously, the mutations L81N and I88N had an even more pronounced impact on the structure of the DENV2C protein, leading to a loss of 58% and 41% in the CD signal, respectively, compared to the WT protein (Fig. 1B). To rule out the possibility of protein aggregation during the spectrum acquisition, and miscalculation of final protein concentration, CD and absorbance spectra were acquired simultaneously from 200 to 300 nm to monitor the absorbance at 280 nm while they were acquired (Supplementary Fig. S1). For all spectra, the absorbance values measured at 280 nm were essentially the same and were used to calculate the molar ellipicity [Θ] of the WT DENV2C and its mutant proteins. And, in all samples, it was not observed change in protein concentration. In conclusion, protein aggregation is a very unlikely explanation for the significant differences observed in the CD spectra of the WT DENV2C and its mutant proteins.

### WT DENV2C and the mutant proteins are dimeric and highly folded in solution

**Figure 2:**
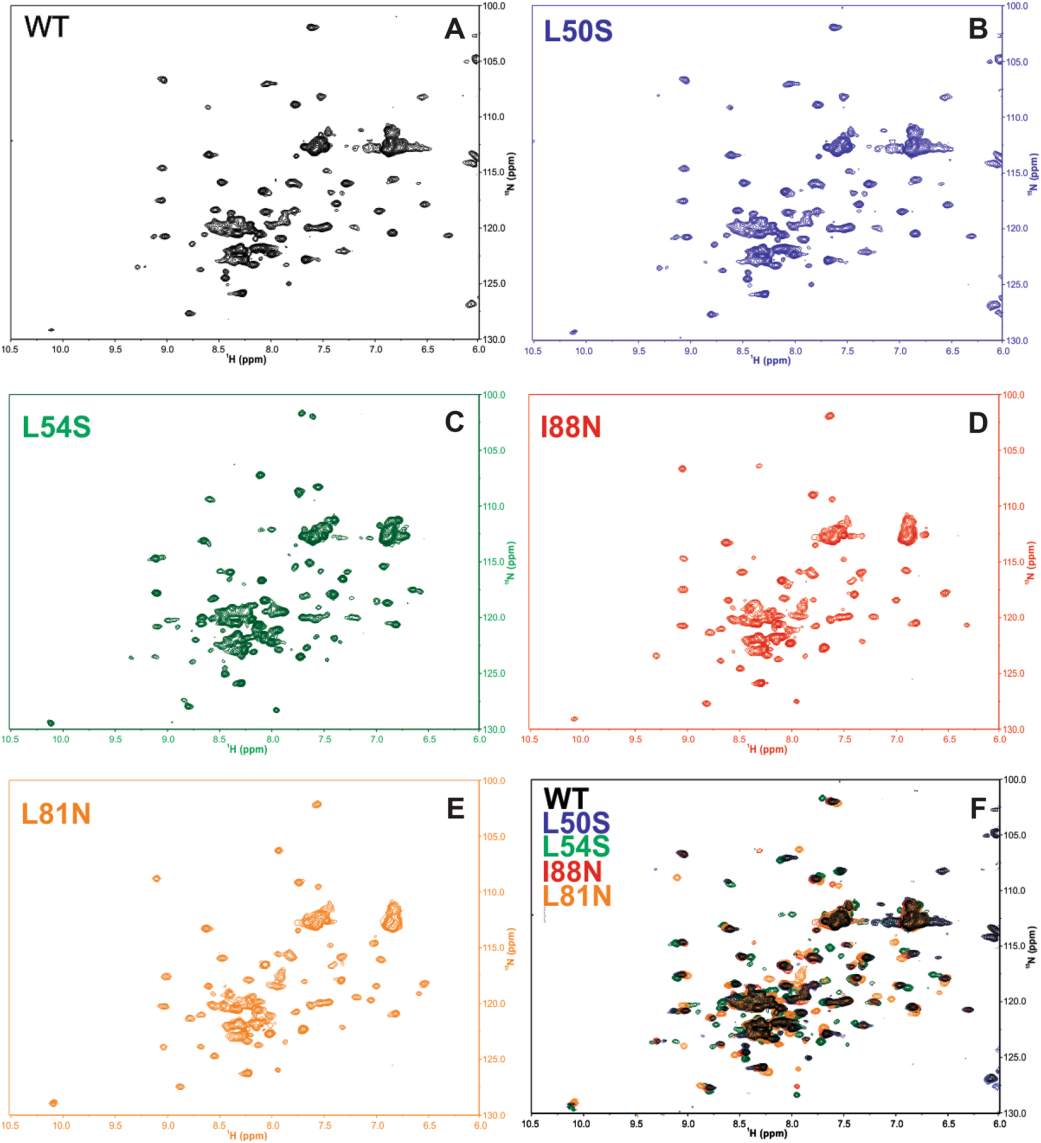
^15^N-HSQC NMR spectra of WT and mutant DENV2C proteins. Samples were diluted in 50 mM sodium phosphate buffer (pH 6.0), 0.2 M NaCl, and the final protein concentration depended on the DENVC construct (see Methods). The experiments were carried out at a 900-MHz spectrometer (Bruker) at 300 K. (**A**) WT DENV2C; (**B**) L50 S DENV2C; (**C**) L54S DENV2C; (**D**) I88N DENV2C; (**E**) L81N DENV2C; and (**F**) spectrum superposition of all five DENV2C proteins.

The tertiary structure of the WT DENV2C and the mutant proteins was evaluated by ^15^N-HSQC NMR spectra (Fig. 2A–F). The spectrum of the WT DENV2C protein was very similar to the ones previously reported^9,12^, confirming that it was obtained in a highly folded state (except the roughly twenty N-terminal amino acid residues that have already been showed to be largely unstructured). Moreover, the large dispersion in the ^15^N and ^1^H chemical shifts observed in the ^15^N-HSQC NMR spectra for the mutant proteins is very strong evidence that no mutation was able to promote DENV2C protein unfolding (at least not detected by NMR experiments). The mutations did change the chemical shift of some amino acids, what can be observed in the superposition of all five ^15^N-HSQC spectra (Fig. 2F). These results are in agreement with the decrease in the secondary structure observed in the CD spectra (Fig. 1B).

**Figure 3:**
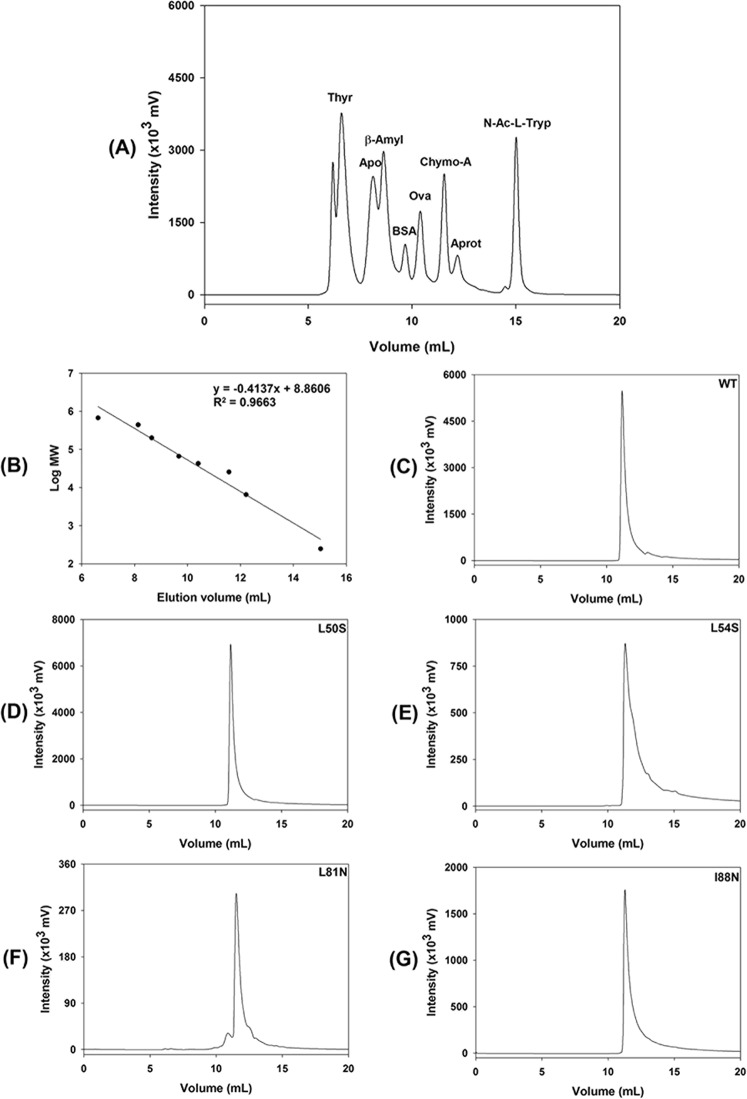
Analysis of the quaternary structure of WT and mutant DENV2C proteins by gel-filtration chromatography using a TSK3000SWXL column. Chromatography was performed at 25 °C in 50 mM sodium phosphate buffer (pH 6.0) containing 0.2 mM NaCl and 0.05% NaN_3_ at a flow rate of 1 mL/min for a duration of 20 min. The injection volume was 200 µL. The protein elution was monitored by a fluorescence detector with excitation at 280 nm and emission at 340 nm. (**A**) Injection of a mixture of standard proteins containing thyroglobulin (Thyr), apoferritin (Apo), β-amylase (β-amyl), bovine serum albumin (BSA), ovalbumin (Ova), chymotrypsinogen A (Chymo-A), Aprotinin (Aprot) and N-acetyl-L-tryptophan (N-Ac-L-Tryp). (**B**) Calibration curve obtained by plotting the elution volume of the standard proteins *versus* the logarithm of the molecular weight (MW). (**C**–**E**), (**F**,**G**) show the injection of 200 µL of 30 µM DENV2C (WT) and single mutants L50S, L54S, L81N and I88N, respectively.

It is well established in the literature that DENV2C is dimeric (apparent molecular mass of 25 kDa) in solution^9,10^. To assess whether the mutations had also affected the quaternary structure of DENV2C, both the WT and mutant proteins were subjected to gel-filtration chromatography using a TSK3000SWXL column that was calibrated with eight molecular-mass standards ranging from 246 to 670,000 Da (Fig. 3A,B). WT DENV2C and the mutants L50S, L54S, L81N and I88N had very similar elution volumes (11.2, 11.2, 11.3, 11.5, and 11.3 mL, respectively) and eluted as a single peak (Fig. 3C–G). These elution volumes are lower than the elution volume of the standard protein chymotrypsinogen A (11.7 mL), which has an apparent molecular mass of 25.6 kDa, confirming that the mutations did not affect the quaternary structure of DENV2C in solution. Altogether, both NMR and gel-filtration chromatography experiments showed that the mutations L50S, L54S, L81N and I88N were not able to promote protein unfolding or dissociation of the dimeric DENV2C.

### Mutations in the α2–α2′ and α4–α4′ dimer interfaces decrease the DENV2C stability

**Figure 4:**
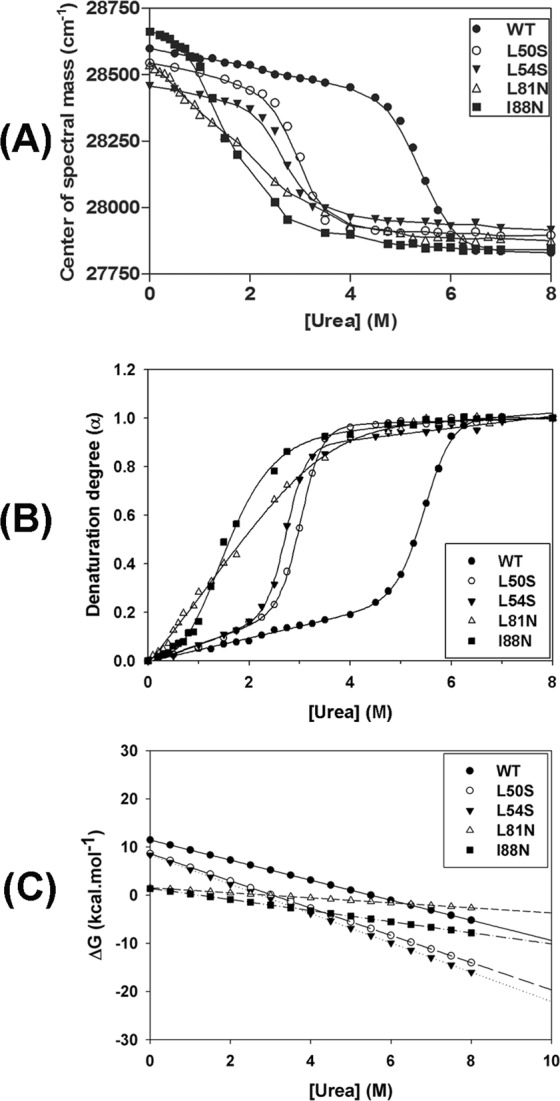
Urea-mediated denaturation of WT and single-point mutant DENV2C proteins was monitored by tryptophan fluorescence and expressed as center of spectral mass (**A**) and degree of denaturation (**B**). Proteins were diluted to a final concentration of 5 µM in 50 mM sodium phosphate buffer (pH 6.0)/0.2 M NaCl containing urea concentrations ranging from 0 to 8 M. Fluorescence emission spectra were recorded using a Cary Eclipse spectrofluorimeter (Varian, Australia) with an excitation wavelength of 280 nm (slit width 5 nm) and emissions were recorded from wavelengths of 300 to 420 nm (slit width 10 nm) at 25 °C. The stability curves of these proteins were obtained by plotting the free energy of unfolding as a function of urea concentration (**C**).

To further characterize the effect of proposed mutations on the structure and stability of DENV2C, a chemical denaturation assay monitored by fluorescence spectroscopy and CD was conducted. Chemical denaturation of the WT and the mutant proteins was performed by a 1-h incubation at room temperature with urea concentrations ranging from 0 to 8 M. Urea-mediated denaturation of protein structure can be detected by a shift in the tryptophan fluorescence spectrum to higher wavelengths as a consequence of increased exposure of tryptophan to solvent. This spectral shift can be monitored by measuring the center of spectral mass (CM). As expected, all proteins showed a decrease in the CM as the urea concentration increased up to 8 M (Fig. 4A); the CM variation obtained for each curve was as follows: ΔCM_WT_ = 769.03 cm^−1^, ΔCM_L50S_ = 648.18 cm^−1^, ΔCM_L54S_ = 541.79 cm^−1^, ΔCM_L81N_ = 661.83 cm^−1^ and ΔCM_I88N_ = 820.95 cm^−1^. The CM was converted into denaturation degree as previously reported^18,19^. Upon evaluation, the urea-denaturation degree showed typical cooperative folding-unfolding transitions with sigmoidal curves (Fig. 4B) for all proteins except the L81N mutant. The curve for the L81N mutant did not show a well-defined initial plateau, while the denaturation curves for all the other proteins showed two well-defined states. The urea-denaturation-degree curves were fitted according to the linear extrapolation method proposed by Pace and Shaw^20^. The stability curves of these proteins were obtained by plotting the free energy of unfolding as a function of urea concentration (Fig. 4C). Unfolding parameters of urea denaturation are shown in Table 1, and the values for ΔG°_H2O_ obtained were as follows: ΔG°_H2O_ (WT) = 11.44 kcal/mol^−1^, ΔG°_H2O_ (L50S) = 8.59 kcal/mol^−1^, ΔG°_H2O_ (L54S) = 8.35 kcal/mol^−1^, ΔG°_H2O_ (L81N) = 1.51 kcal/mol^−1^, and ΔG°_H2O_ (I88N) = 1.38 kcal/mol^−1^. All single-point mutations introduced in DENV2C protein were able to decrease the stability of the WT protein. However, this effect was more pronounced for the L81N and I88N mutations, which led to a great reduction in protein stability, as shown by the low ΔG°_H2O_ values obtained for these mutants. Interestingly, the WT DENV2C protein was shown to be very resistant to chemical denaturation, with the transition phase starting at high urea concentrations of approximately 4.5 M and completely unfolding at approximately 6.0 M; on the other hand, the L50S, L54S, L81N and I88N mutants were already fully denatured at 3 M urea (Fig. 4B). The L50S and L54S mutants initiated their transition phase at urea concentrations of 2 M (Fig. 4B). L81N and I88N were observed to be very sensitive to chemical denaturation. The transition phase of the I88N mutant was initiated at very low urea concentrations (approximately 0.75 M), whereas the L81N mutant exhibited a continuous denaturation process starting at very low initial concentrations of urea.

**Table 1:** Unfolding parameters of urea denaturation of the DENV2C protein and its mutants by fluorescence spectroscopy.

	WT	L50S	L54S	L81N	I88N
U_1/2_ (M)	5.3	2.9	2.7	1.9	1.6
ΔG°_H2O_ (kcal.mol^−1^)	11.44	8.59	8.35	1.51	1.38
*m*	2.08	2.83	3.05	0.52	1.15
**Goodness of Fit**					
R²	0.9998	0.9998	0.9996	0.9994	0.9992

**Figure 5:**
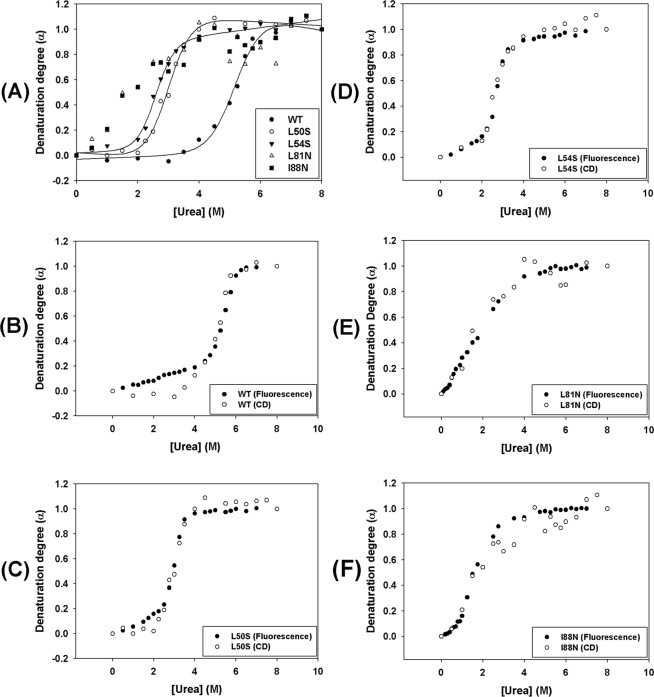
Urea-mediated denaturation of WT and single-point mutant DENV2C proteins was monitored by CD. CD spectra were obtained in a Chirascan (Applied Photophysics, United Kingdom) using quartz cuvettes with 0.1-cm path length at 25 °C. The proteins were diluted in 50 mM sodium phosphate buffer (pH 6.0)/200 mM NaCl containing urea concentrations ranging from 0 to 8 M to a final protein concentration of 10 µM. The molar ellipticity at 222 nm was converted into denaturation degree (α) and was plotted against the urea concentration (**A**). The denaturation degree (α) curves using urea obtained for each protein by CD and tryptophan fluorescence were superimposed and are shown in (**B**–**F**).

The effect of urea-mediated denaturation on the secondary structure of the WT and mutant DENV2C proteins was monitored by CD. The molar ellipticity at 222 nm was used to evaluate the loss of secondary structure as the proteins were subjected to increasing urea concentrations ranging from 0 to 8 M. All proteins showed a complete loss of the CD signal as the urea concentration increased, indicating a loss of α-helical content. After conversion of the values of molar ellipticity at 222 nm into denaturation degree (Fig. 5A), the WT protein and the mutants L50S and L54S showed sigmoidal curves with well-defined initial and final plateaus, as observed in the fluorescence curves. However, the urea-denaturation degree curves determined by CD for the L81N and I88N mutants could not be fitted using the Pace and Shaw method^20^. It is worth mentioning that the urea-denaturation degree curves obtained by both fluorescence and CD spectroscopies for all proteins overlap (Fig. 5B–F). These results strongly suggest that the loss of secondary and tertiary structures occur concomitantly without the detectable presence of folding intermediates.

**Figure 6:**
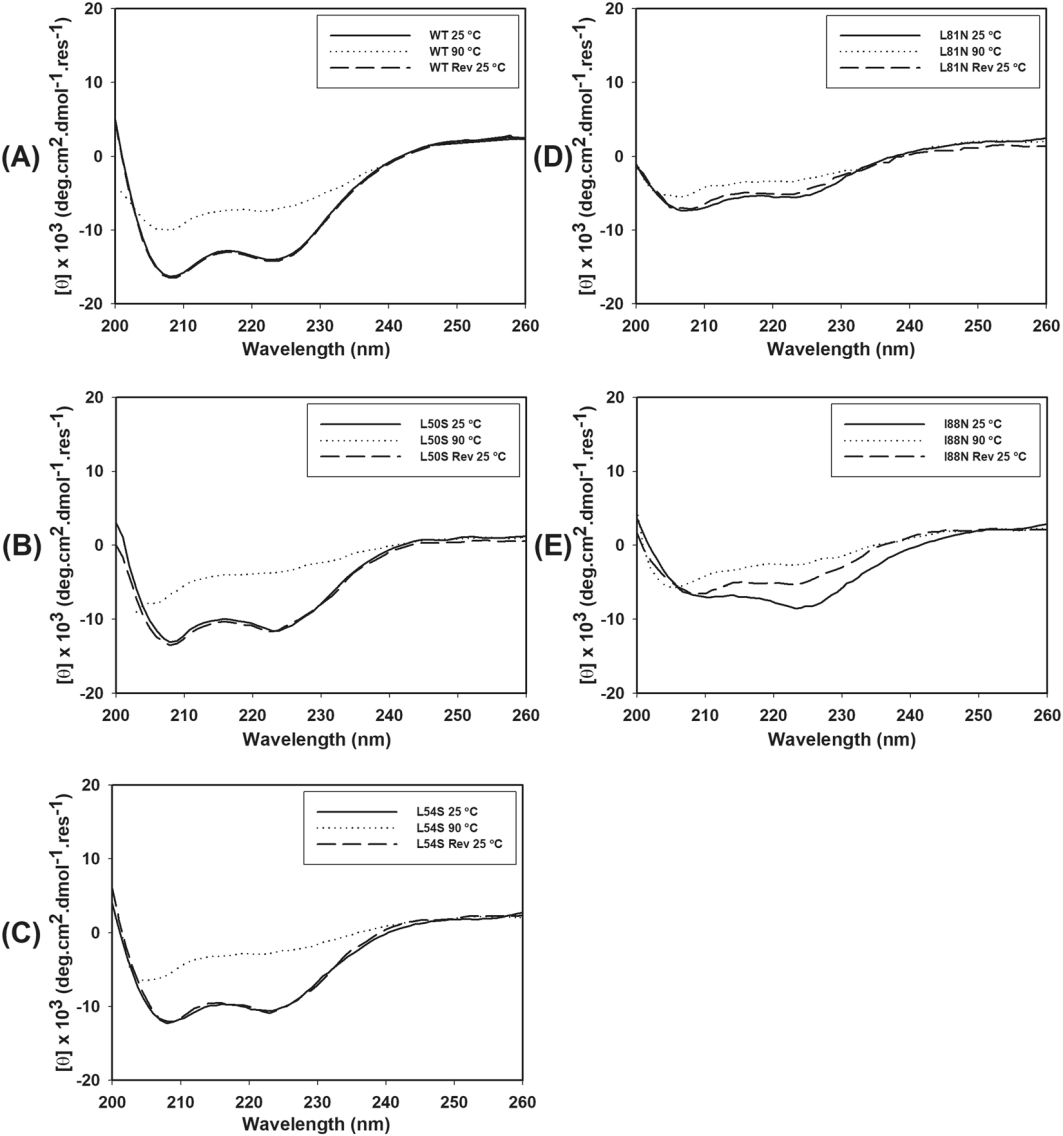
Thermal denaturation of WT and single-point mutant DENV2C WT proteins was monitored by CD. CD spectra were obtained in a Chirascan (Applied Photophysics, United Kingdom) using quartz cuvettes with 0.1-cm path length at 25 °C. The proteins were diluted in 50 mM sodium phosphate buffer (pH 6.0)/200 mM NaCl to a final concentration of 10 µM. Spectra were collected every 5 °C from 20 to 90 °C, with one acquisition at each temperature. The CD spectra of these proteins at 25 °C, 90 °C and after reversion from 90 °C to 25 °C (Rev 25 °C) were plotted from wavelengths of 200 nm to 260 nm.

**Figure 7:**
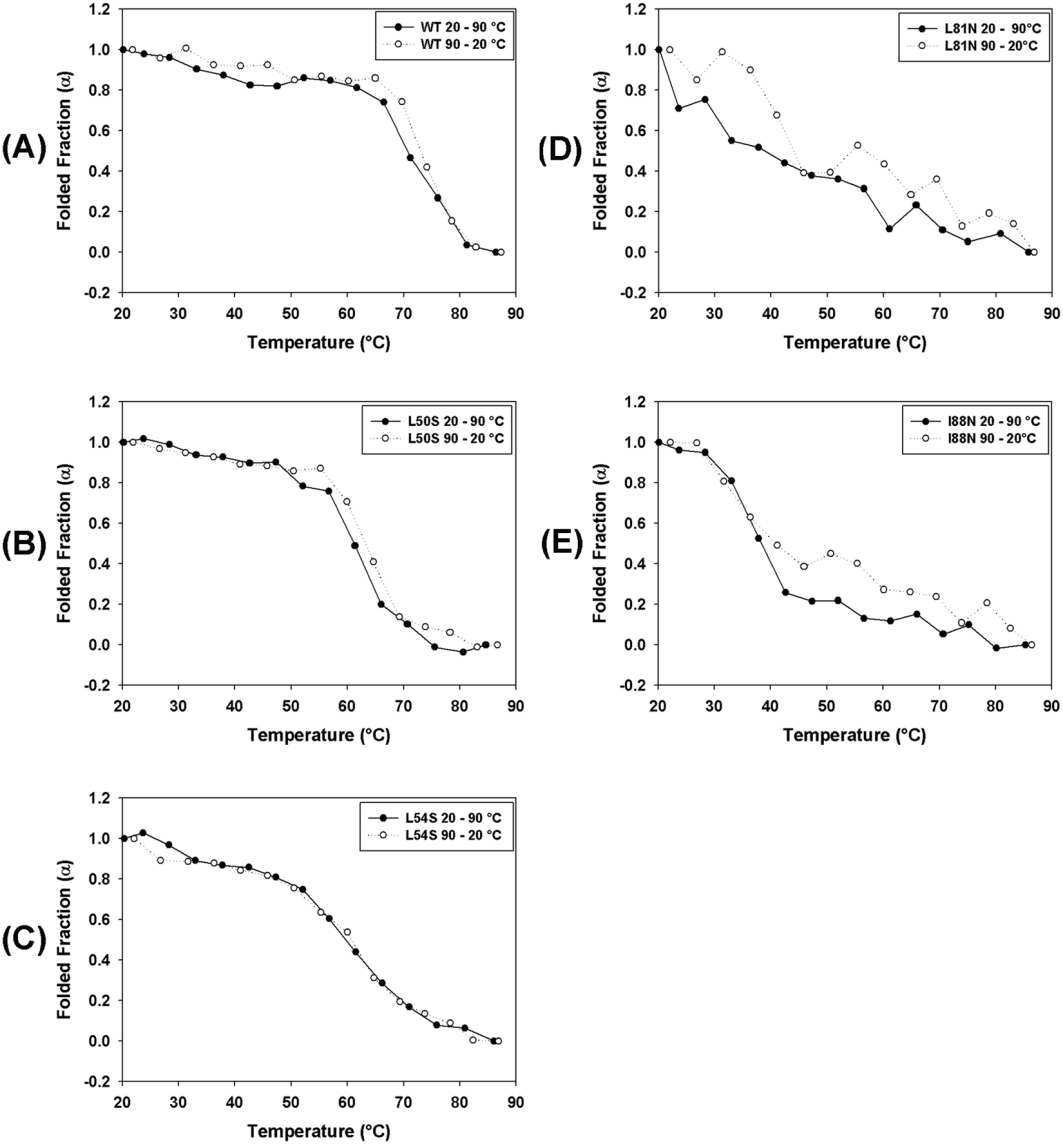
The reversibility of the thermal denaturation of WT and mutant DENV2C proteins were evaluated by circular dichroism. CD spectra were obtained in a Chirascan (Applied Photophysics, United Kingdom) using quartz cuvettes with 0.1-cm path lengths. The proteins were diluted in 50 mM sodium phosphate buffer (pH 6.0)/200 mM NaCl to a final concentration of 10 µM. Spectra were collected every 2 °C from 20 to 90 °C, with one acquisition at each temperature and to evaluate the reversibility of denaturation, spectra were also collected every 2 °C from 90 to 20 °C. The molar ellipticity at 222 nm was used to evaluate the changes in the secondary structure of these proteins promoted by the temperature. The CD signal at 222 nm was converted into a folded fraction (α) value and plotted against temperature. The folded fraction curves acquired from 20 to 90 °C and from 90 to 20 °C were superimposed and are shown in (**A**–**E**).

The stability of WT and mutant DENV2C proteins was also studied by a temperature denaturation assay monitored by CD. The molar ellipticity values were considered to represent the changes promoted by temperature in the secondary structures of the WT and mutant proteins and the reversibility of this process. The CD spectra showed significant reduction in the two negative peaks at approximately 208 and 222 nm for all proteins (Fig. 6). The comparison of the CD spectra indicated that WT, L50S and L54S (Fig. 6A–C, respectively) were more thermostable and that the denaturation process was largely reversible. For the mutants L81N and I88N, the reversion of the temperature from 90 °C to 20 °C led to a loss of the CD signal at 222 nm, an effect that was more evident for the I88N mutant; this observation suggested that the denaturation of the secondary structure of these two mutants was not completely reversed. The reversibility of the thermal denaturation was also evaluated by CD and expressed as denaturation degree. This process was observed to be reversible for all the proteins, as indicated by the overlap of the denaturation degree curves from 20–90 °C and 90–20 °C (Fig. 7). During the spectrum acquisition, it was not observed protein deposition and/or aggregation because the absorbance values at 280 nm for each DENV2C protein did not change significantly with the temperature variation (Supplementary Fig. S2). In addition, the concentration of the samples, which were subjected to temperature denaturation/renaturation, was always measured just before and soon after the spectrum acquisition, and they were not significantly different from each other. In conclusion, the secondary structure of WT DENV2C protein and its mutants is lost because of the temperature denaturation process and not because of protein aggregation/deposition.

### Mutations in the α2–α2′ and α4–α4′ dimer interfaces impair the interaction between DENV2C protein and RNA

**Figure 8:**
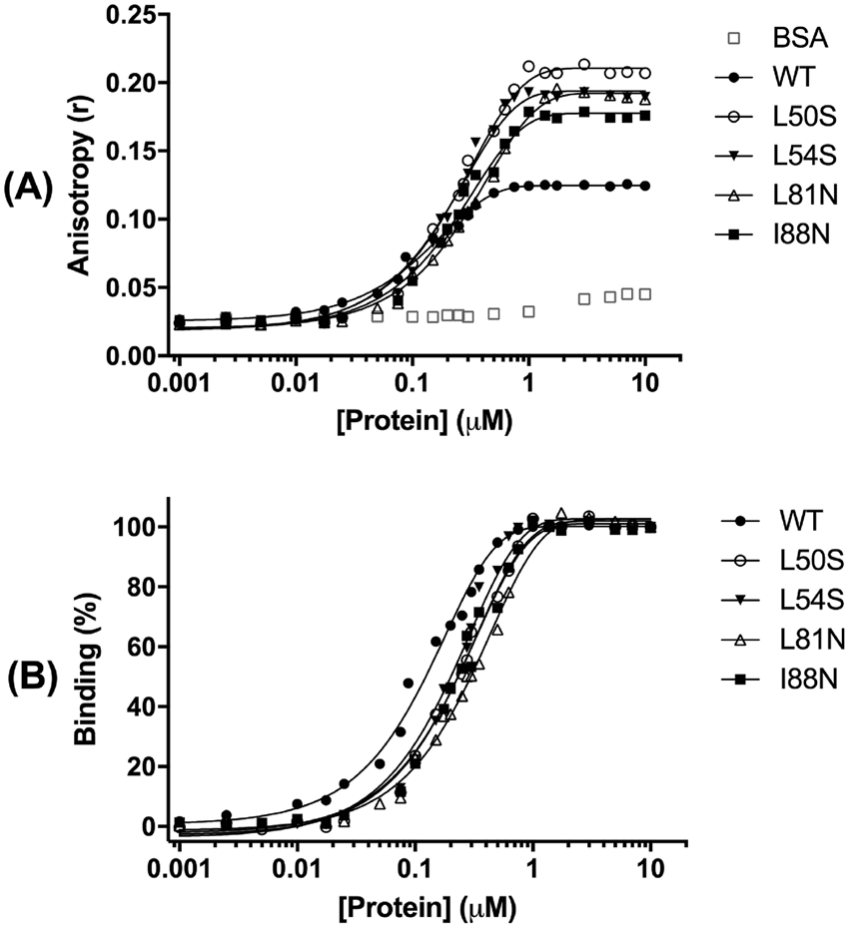
The effect of the DENV2C mutations on capsid-RNA interaction was evaluated. (**A**) Interactions of BSA (negative control), DENV2C WT and L50S, L54S, L81N and I88N mutants with a nonspecific RNA were measured by fluorescence polarization. Reactions were prepared containing 50 nM RNA-FAM, RNase inhibitor (diluted 1:5000), and protein concentrations ranging 0 to 10 µM in 50 mM sodium phosphate/20 mM NaCl, at a reaction volume of 100 µL. The data represent the means of three individual experiments and were fitted by non-linear regression analysis to a one-phase association equation using the least squares method available in GraphPad Prism 7 software. (**B**) The anisotropy value for each protein concentration was also converted into a binding value, which was plotted against protein concentration.

The DENV2C protein interacts with viral RNA to form the capsid. The ability to interact with RNA was measured to determine whether the single-point mutations were able to perturb this interaction. The interaction of the capsid protein and its mutants with a non-specific 20-mer RNA attached to the FAM fluorophore was monitored by fluorescence polarization/anisotropy. The WT and single-point mutant DENV2C proteins were able to interact with non-specific RNA (Fig. 8A), as shown by the increase in anisotropy with an increase in protein concentration. As expected, BSA was not able to interact with RNA-FAM. The data were fitted by nonlinear regression to an exponential one-phase association, and the dissociation constants (Kd) were obtained. The anisotropy curves were also converted into percentages of binding (Fig. 8B). The goodness of fit was evaluated by the parameters presented in Table 2. The Ka values obtained for the interaction of the capsid protein and its mutants with RNA-FAM were as follows: Kd (WT) = 124.8 nM, Kd (L50S) = 228.5 nM, Kd (L54S) = 189.9 nM, Kd (L81N) = 302.1 nM, and Kd (I88N) = 228.3 nM. The Kd values indicate that all the single-point mutations affected the ability of the capsid protein to interact with RNA. The single-point mutants L50S, L54S and I88N exhibited very similar Kd values compared to the L81N mutant, which showed the lowest affinity for RNA among all the proteins. All mutations affected the interaction of the capsid protein with RNA; however, the L81N mutation had a more pronounced effect, probably because this residue is more effectively involved in RNA interaction and/or dimer stabilization.

**Table 2:** Dissociation constant (K_d_) of DENV2C protein and its single mutants to FAM-labeled RNA.

	WT	L50S	L54S	L81N	I88N
K_d_ [nM]	124.8	228.5	189.9	302.1	228.3
**95% Confidence Intervals**					
K_d_ [nM]	114.4 to 137.2	209.0 to 252.0	178.7 to 224.2	282.0 to 325.4	206.7 to 254.9
**Goodness of Fit**					
Degrees of Freedom	21	20	22	22	22
R²	0,9954	0,9955	0.9914	0.9964	0,9923
Absolute Sum of Squares	0.0001757	0.0006295	0.001021	0.0003745	0.0007309
Sy.x	0.002892	0.00561	0.006813	0.004126	0.005764
Number of analyzed points	24	23	25	25	25
Number of points	28	28	28	28	28

Non-linear regression analysis with one-phase association equation using the least squares fitting method available in GraphPad Prism 7 software.

## Discussion

The single-point mutations in DENV2C protein were localized to the α2 helix (L50S and L54S), reproducing mutants previously described by Samsa *et al*.^15^, and to the α4 helix (L81N and I88N), reproducing mutants described by Patkar *et al*.^17^. Samsa *et al*. (2009) showed that the DENV2C protein accumulates in the cytoplasm of DENV-infected cells at the boundaries of lipid droplets and that there is an increase in the number of lipid droplets per cell during DENV infection; these data suggest a relation between viral replication and lipid-droplet metabolism. In addition, pharmacological intervention at the lipid droplet also reduced viral replication. Mutations at the L50 and L54 residues of DENV2C were described to interfere with capsid integration into lipid droplets, impairing viral particle assembly. Patkar *et al*. (2007) predicted the structure of the YFV capsid using a homology model based on the DENV2C structure^17^. Mutations at residues 81 and 88 impaired viral assembly. This study showed that the single-point mutations L78N, M92N, and L95N resulted in fairly small effects on YFV C protein activity, as measured by luciferase assay. However, the L81N and V88N mutations promoted a significant reduction in this activity compared to the WT protein, suggesting that this decrease might be a result of the destabilization of the capsid dimer.

The α2 and α4 helices are part of the major area of the dimerization surface, which is composed of two antiparallel helices, namely, α2-α2′ and α4-α4′. The interface α2-α2′ is part of an internal hydrophobic region on the DENV2C protein, which has been proposed to be the region interacting with membranes. The interface α4-α4′ is held by hydrophobic interactions between the side chains of the residues I78, L81, I88, L92 and L95 on one monomer with their counterparts on the antiparallel monomer. The α4-α4′ region is rich in basic residues and has been proposed to interact with RNA^10^.

In this study, WT DENV2C and its mutants were expressed and purified at high purity (Fig. 3) and were shown to fold as α-helical proteins (Fig. 1), as expected for *Flavivirus* capsid proteins^9^. All the single-point mutations affected the secondary structure of the capsid protein, as was demonstrated by the decrease of the CD signal (Fig. 1B). However, mutations at residues of helix α4 had a greater effect on the secondary structure than that observed with mutations at residues of helix α2. We also attempted to generate double mutants (L50S/L54S and L81N/I88N), which were demonstrated to be very unstable, fully aggregating during purification (data not shown).

The tertiary and quaternary structures of the capsid and its mutants were evaluated by NMR and gel-filtration chromatography experiments. The NMR experiments clearly demonstrated typical ^15^N-HSQC spectra of folded protein for WT DENV2C and the mutant proteins with broad chemical shift dispersions in both dimensions (Fig. 2). Nonetheless, the mutations did change the chemical shifts of some amino acids, confirming their major effect in the secondary structure of the protein. Moreover, it is very likely that the mutations also affected the tertiary structure to some extent, but the overall native-like topology of the mutant DENV2C proteins were maintained. It is worthy mentioning that if any of the mutations studied here caused the DENV2C protein unfolding, the ^1^H chemical shifts in the ^15^N-HSQC spectra would appear in a very narrow range (about 1 ppm)^21,22^. It was also confirmed that the WT DENV2C and the mutant proteins were primarily eluted from the TSK3000SWXL column as dimers (Fig. 3), corroborating the data obtained for the DENV capsid protein produced by chemical synthesis, which also showed an elution profile composed primarily of dimers by gel-filtration chromatography^23^. In conclusion, the change in the secondary structure, protein stability and RNA interaction observed in the mutations in the α2-α2′ and α4-α4′ dimer interfaces of DENV2C cannot be explained by protein unfolding.

The structural stability of the capsid protein and its mutants was studied by a chemical denaturation assay monitored by fluorescence spectroscopy and CD as well as by a thermal denaturation assay monitored by CD. All single-point mutations introduced in the DENV2C structure promoted a reduction in protein stability, an effect that was more pronounced in the L81N and I88N mutants, as indicated by the low ΔG°_H2O_ value obtained for these mutants. The WT protein was demonstrated to be very stable to urea denaturation, showing a U_1/2_ value of approximately 5.3 M (Table 1). Analysis of the urea-denaturation degree curves obtained by CD and fluorescence spectroscopy demonstrated that these curves overlapped; therefore, it was possible to conclude that the changes in secondary structure promoted by each concentration of urea are reflected in the changes observed in the tertiary structure. These curves did not show inflections and were observed to be typically cooperative with two-state unfolding transition, suggesting the absence of unfolding intermediates (Fig. 5). Only the L81N mutant did not present a defined initial plateau in the urea-denaturation degree curves; this process started at initial concentrations of urea and seemed to be continuous until the protein reached the unfolded state above urea concentrations of 4 M (Fig. 4B).

The temperature denaturation data obtained by CD showed that the WT protein is highly resistant to heat, showing an α value of approximately 0.5 at high temperatures from 70 to 75 °C (Fig. 7). The mutations in DENV2C protein also significantly affected the protein stability to changes in temperature. The L50S and L54S mutants exhibited α values of 0.5 at approximately 60 °C. L81N and I88N mutants were very unstable to changes in temperature, already exhibiting an α value of 0.5 at temperatures from 30 to 40 °C. In addition, the reversibility of thermal denaturation was also evaluated, and this process was shown to be reversible for all analyzed proteins (Fig. 7). Our data indicate that the capsid protein is thermostable and exhibits thermal flexibility, allowing this protein to refold after exposure to high temperatures (Figs 6 and 7).

The capsid protein has a primary structural role in the protection of the viral genome during transition between environments, such as entry into and exit from host cells^24^. This protein needs to protect the RNA against exposure to chemical hazards in the environment^25^. Our findings regarding the high chemical and thermal structural stability of DENV2C is in agreement with the requirements of this protein to perform its protective role.

A few studies have tried to evaluate the interaction of DENV2C with RNA. The role of DENV2C as an RNA chaperone was studied, and this protein was shown to facilitate the formation of the typical hammerhead structure of RNA^26^. Another previous study also assayed the ability of the DENV capsid to interact with RNA; the DENV capsid protein and the double mutant (L50S-L54S) interacted with the DENV 5′-UTR RNA (labeled with ^32^P) with a high affinity, but no significant differences were observed in the Kd values obtained when comparing the WT protein and the double mutant (Kd_WT_ = 22 nM and Kd_L50S-L54S_ = 20 nM)^15^.

In this study, the interaction of the capsid protein was measured with non-specific 20-mer RNA by fluorescence polarization. The effect of mutations at residues from the α2-α2′ and α4-α4′ interfaces on capsid–RNA interaction was also studied. All the single-point mutations affected the ability of the capsid protein to interact with RNA, as observed in the Kd values obtained (Table 2). The Kd values of the L50S, L54S and I88N mutants were very close compared to that for the L81N mutant, which showed the lowest affinity for RNA among all the mutants, probably because this residue could be more effectively involved with RNA interaction and/or dimer stabilization. It is worth mentioning that the RNA-DENV2C Kd was only possible to be calculated because of type of experimental design in which a very short labeled-RNA (20 mer) at a very low concentration (in a nM range) was used in the experiment avoiding complex aggregation. It was observed a clear plateau up to 10 µM of protein concentration, strongly suggesting a bimolecular binding curve. It is worth mentioning that we did not aim to calculate the absolute affinity constant but rather to analyze the relative effect of each point mutation on the RNA-DENV2C complex formation.

As observed in the fluorescence polarization graph (Fig. 8A), the anisotropy values at the plateau for the WT DENV2C protein were lower than those obtained for the single mutants. The fluorescence anisotropy measures the rotational diffusion of a molecule, and as a result, the anisotropy will be sensitive to any factor that changes the rotational diffusion. Factors such as the size and shape of rotating biomolecules can affect the rate of rotational diffusion^27^. The observed differences in the plateaus in Fig. 8A could be associated with the reduction of the structural stabilities of these mutant proteins, the change in shape, or the increased flexibility of the protein backbone, which could lead to a decrease in rotational diffusion and consequently increase the anisotropy. Another possible explanation could be that the single mutations increase the flexibility of the protein, allowing the binding of more molecules of RNA at the same binding site in these proteins, leading to a reduction of rotational diffusion and an increase in anisotropy.

In conclusion, we showed that mutations in the α2-α2′ and α4-α4′ dimer interfaces of DENV2C affect the structural stability and reduce RNA-capsid interaction. However, the mutation at the residue L81 was the one that had the greatest effect on protein stability and on the interaction of the protein with RNA. Our data support the observation of Patkar *et al*.^17^, who showed that point mutations at the L81 and I88 residues of the α4 helix were those that most affect viral assembly, probably because these mutations can affect the affinity for RNA.

It is clear that the capsid protein plays an important role in *Flavivirus* biology as it assembles to form the nucleocapsid and functions in the replication cycle; therefore, this protein is considered a potential antiviral target^28^. Our results highlight the importance of the α4-α4′ dimer interface of DENV2C, which can be a target for drug design in the future.

## Methods

### Expression and purification of WT and mutant DENV2C proteins

The *DENV2C* gene encoding the first 100 amino acids residues of the DENV2C protein was cloned into the pET21a vector. The single-point mutations (L50S, L54S, L81N and I88N) were introduced into the *DENV2C* gene by site-directed mutagenesis using inverse PCR. *E*. *coli* cells (CodonPlus-pRIL strain, Novagen, USA) were transformed with either WT (pET21a-DENV2C) plasmid or with a plasmid carrying a single-point mutation. The protein expression was carried out according the protocol published previously^9,14^. For the NMR experiments, the plasmid-transformed bacteria were grown in minimal medium containing ^15^NH_4_Cl salt as nitrogen source, following the protocols already described elsewhere^10,12^. NMR experiments were performed at 300 K with 500 μL protein sample dissolved in 50 mM phosphate buffer, pH 6.0, and 0.2 M NaCl in Bruker Avance III 900 MHz spectrometer, equipped with triple-resonance (^1^H, ^13^C, ^15^N) probes installed at the National Center for Bioimaging (CENABIO-UFRJ). The final protein concentration was carried out up to a value that aggregation was avoided, therefore, each DENV2C sample was concentrated at different values as follows: WT (180 µM); L50S (170 µM); L54S (185 µM); I88N (144 µM) and L81N (35 µM). NMR spectra were processed and analyzed using TopSpin 3 (Bruker) and MesReNova (MestreLab Research S. L.) softwares, respectively.

### Gel-filtration chromatography

The tertiary and quaternary structures of the WT and mutant DENV2C proteins were evaluated by gel-filtration chromatography using a TSK3000SW_XL_ column (Tosoh, Japan). The column was coupled to a Shimadzu HPLC and equilibrated using 50 mM sodium phosphate buffer (pH 6.0) containing 200 mM NaCl and 0.05% NaN_3_ (equilibration buffer)_._ The calibration curve was obtained by using a mixture of the following standard proteins prepared in the equilibration buffer: 1 mg/mL thyroglobulin (Thyr), 1 mg/mL apoferritin (Apo), 50 µg/mL β-amylase (β-amyl), 50 µg/mL bovine serum albumin (BSA), 100 µg/mL ovalbumin (Ova), 100 µg/mL chymotrypsinogen A (Chymo-A), 2 mg/mL aprotinin (Aprot) and 4 µg/mL N-acetyl-L-tryptophan (N-Ac-L-Tryp). DENV2C (WT) and single-point mutants (L50S, L54S, L81N and I88N) of DENV2C were diluted to 30 µM in equilibration buffer. Chromatography was performed at 25 °C with a flow rate of 1 mL/min and an injection volume of 200 µL. Protein elution was monitored by a fluorescence detector with excitation at 280 nm and emission at 340 nm. A calibration curve was obtained by plotting the elution volume of each standard protein *versus* the logarithm of the molecular weight of the protein.

### Thermal and chemical denaturation monitored by circular dichroism

Circular dichroism (CD) analysis was performed to evaluate the effects of temperature and urea concentration on the secondary structures of WT and mutant DENV2C proteins. CD spectra were acquired on a Chirascan (Applied Photophysics, United Kingdom) using quartz cuvettes with either 0.01 cm or 0.1 cm path lengths depending on the experiment to be performed and on the concentration of the protein to be assayed (0.01 cm for 30 µM protein or 0.1 cm for 10 µM protein). The monochromator was set at 260 nm with a bandwidth of 1 nm. Spectra were recorded from 185 to 260 nm at a speed of 1 nm/s. For thermal denaturation curves, the proteins were diluted in 50 mM sodium phosphate buffer (pH 6.0)/200 mM NaCl to a final concentration of 10 µM. Spectra were acquired every 5 °C from 20 to 90 °C, with one acquisition at each temperature. The reversibility of the thermal denaturation curves was assayed with the spectra collected every 5 °C from 90 to 20 °C, with one spectrum acquisition at each temperature. For the chemical denaturation curve, the proteins were diluted to a final concentration of 10 µM with 50 mM sodium phosphate buffer (pH 6.0) containing 200 mM NaCl and 0–8 M urea. The denaturation reaction was incubated for 1 h before spectrum acquisition. The final spectra were converted into molar ellipticities after buffer and baseline subtractions. Molar ellipticity [θ] measurements were calculated as previously reported^18,19^.

### Chemical denaturation monitored by tryptophan fluorescence spectroscopy

The effects of urea on the tertiary structures of WT and single-point mutant DENV2C proteins were also measured by fluorescence spectroscopy, wherein the intrinsic fluorescence from the single tryptophan residue present in these proteins was monitored. Urea-mediated denaturation assays were performed at 25 °C with the proteins diluted to a final concentration of 5 µM in 50 mM sodium phosphate buffer (pH 6.0) containing 200 mM NaCl and 0–8 M urea. The spectra were acquired after 1 h of incubation. Fluorescence emission spectra were recorded using a Cary Eclipse spectrofluorimeter (Varian, Australia) with an excitation wavelength of 280 nm (slit width 5 nm); the emission was recorded at wavelengths of 300 to 420 nm (slit width 10 nm). The center of spectral mass ( < ν > ), the denaturation degree (α) and the free energy change (ΔG) were calculated as previously reported^18,19^.

### Fluorescence polarization assay

The interactions of the WT and mutant DENV2C proteins with RNA was measured by fluorescence polarization using a non-specific RNA attached to the FAM fluorophore (FAM-5′-AACAGUUUCCUUUUCUCUCC), which was manufactured by TriLink Biotechnologies (USA). Reactions containing 50 nM RNA-FAM; RNase inhibitor (diluted 1:5000); and protein at concentrations ranging 0 to 3 µM in 50 mM sodium phosphate (pH 6.0) and 20 mM NaCl were prepared with a reaction volume of 100 µL. The reaction buffer was prepared using RNase-free water. Bovine serum albumin (BSA) was used was a negative control. The filters selected for FAM fluorescence were 490 nm (excitation wavelength) and 520 nm (emission wavelength) in the polarization mode, with a 515-nm cut-off filter and a photomultiplier tube set to high sensitivity. The assay was prepared in a 96-well opaque plate (Greiner Bio-One, USA), which was shaken for approximately 30 s and incubated in the dark at 25 °C for 10 min. The measurements were recorded using a SpectraMax M5 plate reader (Molecular Devices, USA) with one hundred readings per well. The data represent the means of three individual experiments and were fitted by non-linear regression analysis to the one-phase association equation using the least squares method available in GraphPad Prism 7 software. The anisotropy value (*r*) for each protein concentration was converted into binding percentage using eq. 1.

1 $${\rm{B}}=([r]-{[r]}_{{\rm{f}}})/({[r]}_{{\rm{b}}}\mbox{--}{[r]}_{{\rm{f}}})$$

In this equation, B is binding; [*r*] is the experimental anisotropy; [*r*]_f_ is the anisotropy for the free ligand; and [*r*]_b_ is the anisotropy for the fully bound ligand.

## Supplementary information

supplementary Information

## Data Availability

All data generated or analyzed during this study are included in this published article.
