# Supplementary material for: Mutations in the dimer interfaces of the dengue virus capsid protein affect structural stability and impair RNA-capsid interaction
Source: Sci Rep. 2019 Feb 26;9:2829. doi: 10.1038/s41598-019-39185-3 (PMC6391532; doi:10.1038/s41598-019-39185-3)
Supplement: Supplementary file 1 — supplementary Information [file 41598_2019_39185_MOESM1_ESM.pdf]

Mutations in the dimer interfaces of the dengue virus capsid protein affect structural stability and impair RNA-capsid interaction

Janaina Figueira-Mansur[1], Estefania A. Aguilera[1], Rafael M. Stoque[1], Gustavo T. Ventura[1] and Ronaldo Mohana-Borges[1]*.

[1]Laboratório de Genômica Estrutural, Instituto de Biofísica Carlos Chagas Filho, Universidade Federal do Rio de Janeiro, Rio de Janeiro, Brazil

*Corresponding author

E-mail: Mohana@biof.ufrj.br

Telephone: +55-21-39386538

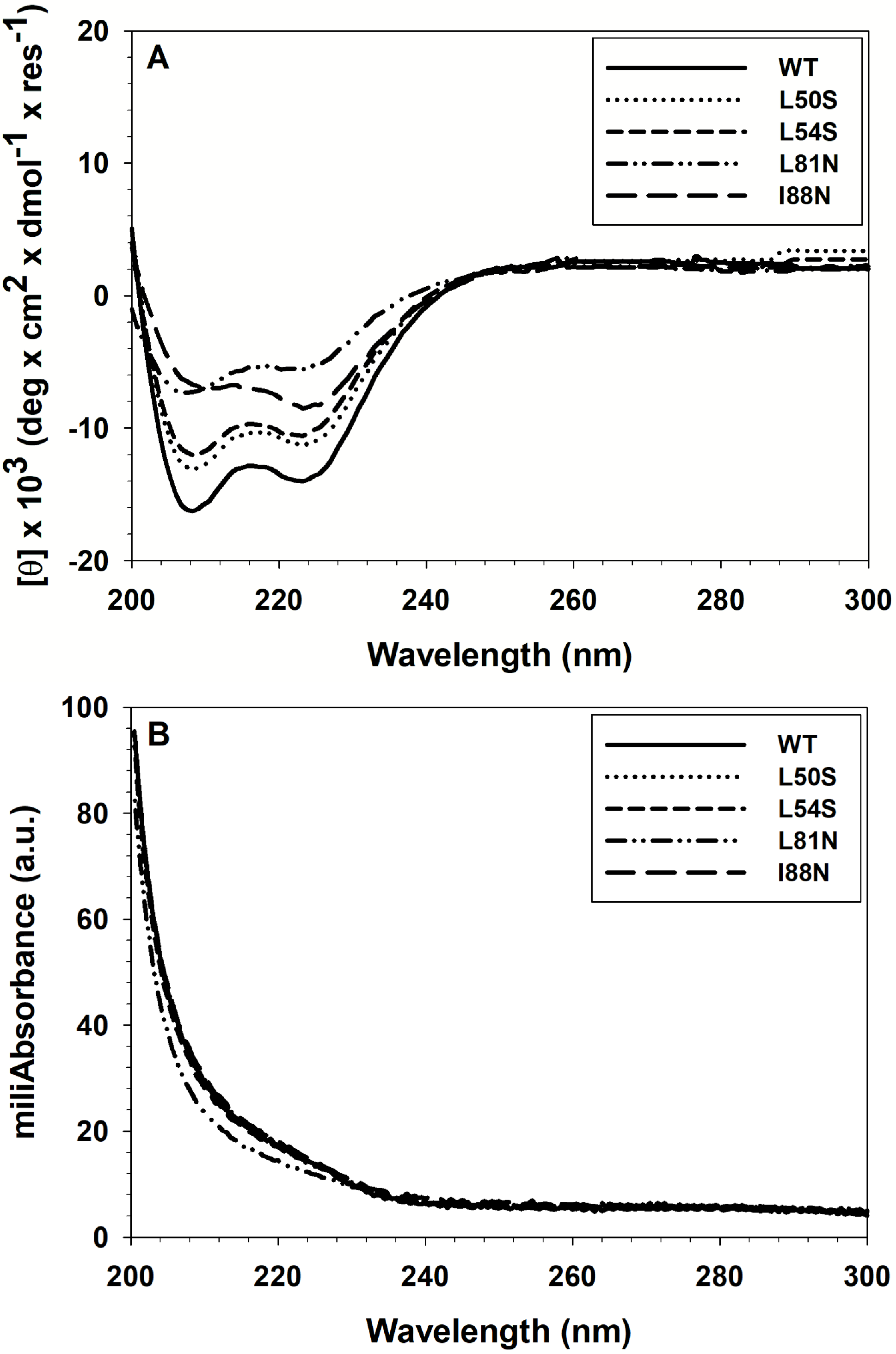

Supplementary Fig. S1 - Analysis of the secondary structure of WT and single-point mutant (L50S, L54S, L81N and I88N) DENV2C proteins by circular dichroism. (A) CD spectra were obtained in a Chirascan (Applied Photophysics, United Kingdom) using quartz cuvettes with a 0.01-cm path length at 25°C. The proteins were diluted in 50 mM sodium phosphate buffer (pH 6.0)/ 200 mM NaCl to a final concentration of 30 μM. Final spectra were the averages of triplicates after buffer and baseline subtractions and were plotted from wavelengths of 200 nm to 300 nm. (B) Absorbance spectra of WT and single-point mutant (L50S, L54S, L81N and I88N) DENV2C proteins as the CD spectra were being collected.

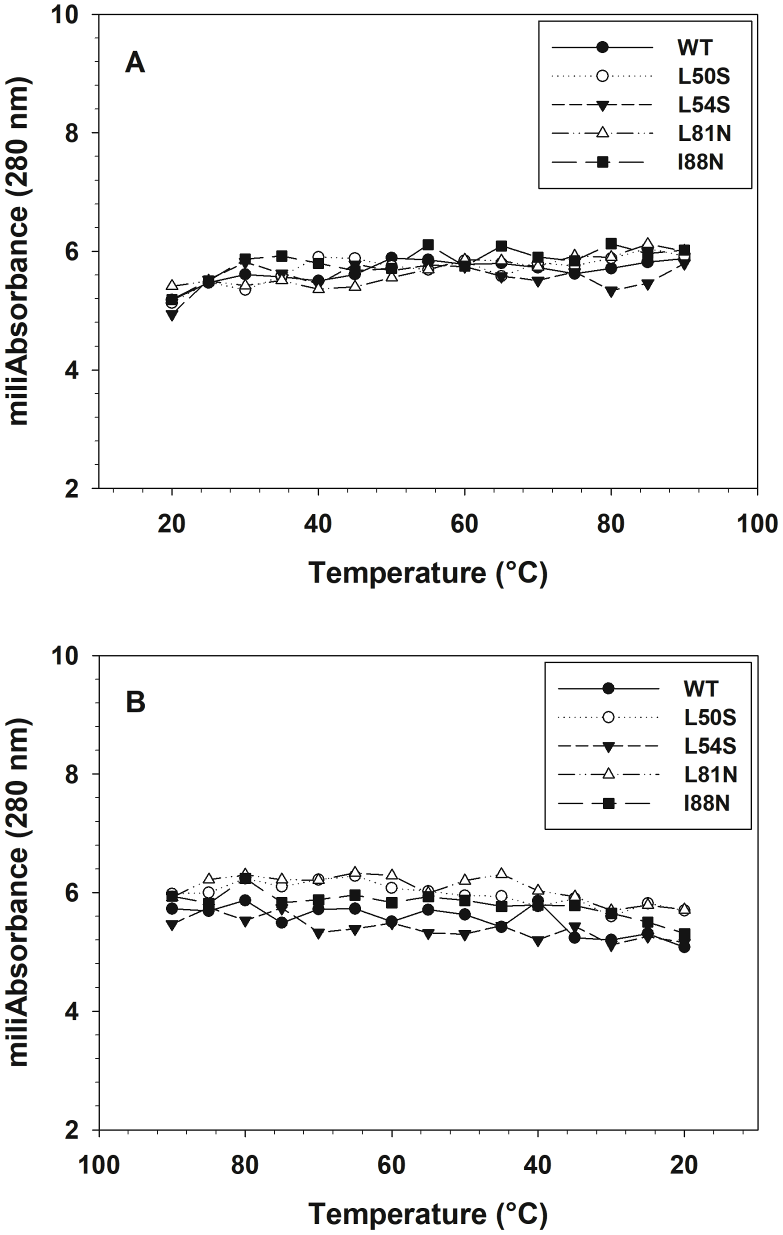

Supplementary Fig. S2 - Thermal denaturation of WT and mutant DENV2C proteins monitored by absorbance at 280 nm. Absorbance spectra were obtained in a Chirascan (Applied Photophysics, United Kingdom) using quartz cuvettes with 0.1-cm path lengths. The proteins were diluted in 50 mM sodium phosphate buffer (pH 6.0)/200 mM NaCl to a final concentration of 10 μM. Spectra were collected every 2°C from 20 to 90°C (A), with one acquisition at each temperature and to evaluate the reversibility of denaturation, spectra were also collected every 2°C from 90 to 20°C (B). The Absorbance at 280 nm was used to evaluate potential protein aggregation/deposition promoted by temperature.
